# Nanoparticles in Clinical Trials: Analysis of Clinical Trials, FDA Approvals and Use for COVID-19 Vaccines

**DOI:** 10.3390/ijms24010787

**Published:** 2023-01-02

**Authors:** Eugenia D. Namiot, Aleksandr V. Sokolov, Vladimir N. Chubarev, Vadim V. Tarasov, Helgi B. Schiöth

**Affiliations:** 1 Department of Neuroscience, Functional Pharmacology, Uppsala University, 75237 Uppsala, Sweden; 2 Advanced Molecular Technology, Limited Liable Company (LLC), 354340 Moscow, Russia; 3 Unit of Functional Pharmacology and Neuroscience, Department of Surgical Sciences, Uppsala University, 75124 Uppsala, Sweden

**Keywords:** nanoparticles, clinical trials, liposomes, clinicaltrials.gov

## Abstract

Nanoparticles are heterologous small composites that are usually between 1 and 100 nanometers in size. They are applied in many areas of medicine with one of them being drug delivery. Nanoparticles have a number of advantages as drug carriers which include reduced toxic effects, increased bioavailability, and their ability to be modified for specific tissues or cells. Due to the exciting development of nanotechnology concomitant with advances in biotechnology and medicine, the number of clinical trials devoted to nanoparticles for drug delivery is growing rapidly. Some nanoparticles, lipid-based types, in particular, played a crucial role in the developing and manufacturing of the two COVID-19 vaccines—Pfizer and Moderna—that are now being widely used. In this analysis, we provide a quantitative survey of clinical trials using nanoparticles during the period from 2002 to 2021 as well as the recent FDA-approved drugs (since 2016). A total of 486 clinical trials were identified using the clinicaltrials.gov database. The prevailing types of nanoparticles were liposomes (44%) and protein-based formulations (26%) during this period. The most commonly investigated content of the nanoparticles were paclitaxel (23%), metals (11%), doxorubicin (9%), bupivacaine and various vaccines (both were 8%). Among the FDA-approved nanoparticle drugs, polymeric (29%), liposomal (22%) and lipid-based (21%) drugs were the most common. In this analysis, we also discuss the differential development of the diverse groups of nanoparticles and their content, as well as the underlying factors behind the trends.

## 1. Introduction

All currently existing diseases can be described in terms of changes at the molecular level, which served as one of the incentives for the development of nanomedicine [1]. Traditionally, nanomedicine is associated with the development of drug delivery systems, primarily used in the treatment of cancer [2,3,4,5]. However, most of the new research points to the versatility of nanoparticles’ properties, which can display their own therapeutic properties [5,6,7]. The greatest successes have been achieved in the field of oncology, for example, a solution of cytarabine and daunorubicin, which have two different pharmacological properties, but are able to coexist inside the nanoparticle [6,8]. Another prime example of nanomedicine application in oncology might be a newly proposed system of zinc oxide nanoparticles for glioblastoma treatment [9]. There have been significant advances in other areas of medicine, such as imaging or immunology [10,11,12]. One of the relatively new magnetic systems based on iron nanoparticles showed greater efficacy and imposes less danger compared to traditional methods for assessing sentinel lymph nodes biopsy or other targeted drug delivery [13,14]. Thus, nanomedicine is an excellent tool for improving the effectiveness of existing therapies and creating new drugs.

Nanoparticles, being one of the most important sections of nanomedicine, are small composites whose size varies from about 1 to 100 nm. Nanotechnology is currently applied in almost all areas of medical sciences, including pharmacology. Very often, conventional drugs have many side effects or low bioavailability, which significantly limits their use. Therefore, one of the important and promising areas is the development of nanoparticles that serve as a method of drug delivery. This is due to the fact that when creating a drug carrier, additional ligands can be attached on it, which would provide greater specificity of the particle to reach certain tissues or to reduce the toxic effect of drugs [15]. The use of nanoparticles as drug carriers has enabled the improvement in drug kinetics, for example, by increasing the circulation time and modulating the interactions between tissues and the drug [16].

Nanoparticles are often classified into three broad groups: organic, inorganic and carbon-based structures. Sometimes the latter group is not distinguished separately. Organic structures are those that are created from natural substances (e.g., albumin). Such nanoparticles may be favorable because of their biocompatibility and low antigenic effects, especially in the case of liposomes [17,18]. Typical representatives of the inorganic group are metals and metal oxides. Occasionally, silica, lanthanide nanoparticles, quantum dots, and other rare nanoparticles are grouped with other inorganic structures. Another way to classify nanoparticles is by dividing them into protein, polymeric, metallic, liposomal, lipid-based and some other structures. Depending on the structure of the nanoparticle, its application also changes. Metal particles can be used as photothermal therapeutic agents and in the diagnosis of neoplasms, while albumin is a promising platform for improving the solubility of, for example, docetaxel and paclitaxel [19]. For instance, silver nanoparticles were found to display greater dispersion and stability which renders them as promising candidates in nanomedicine [20]. Furthermore, there has been an emerging interest in magnetic nanoparticles, which can be applied both for diagnosis and the treatment of a disease. The main idea is to control the site-specific delivery of such nanoparticles just by using the magnetic field [21,22].

Two large groups that are frequently mentioned in various types of research are polymeric particles and liposomes. The first one consists of a polymer frame with which drugs are connected through special linker structures. Polymeric particles can be created from both natural (gelatin) and synthetic chemical substances (polylactides). The presence of a hydrophobic core makes it possible to increase the solubility of drugs, and the hydrophilic membrane significantly increases the circulation time of the complex in the blood. Polymeric particles have a tremendous breadth of application both in diagnostics and in the treatment of diseases, making it possible to additionally assess the efficacy of the existing treatment [23]. Liposomes are specific spherical vesicles, chemically similar to cell membranes and are characterized by a low frequency of allergic reactions and systemic toxicity in response to ingestion [24]. The main difficulty in using liposomes as drug carriers is the ability of the reticuloendothelial system to absorb injected liposomal vesicles. Various coatings are used as a way to escape the cells of the immune system, the most popular of which is polyethylene glycol (PEG). The most successful example of PEGylated liposomes, or stealth liposomes, is Doxil which was approved in 1995 against acquired immunodeficiency syndrome (AIDS)-related Kaposi’s sarcoma [24,25]. However, nanomedicine is not defined by only polymeric and liposomal particles. Recent research suggests new promising and complex formulations, for instance, hydrogel nanoparticles especially in terms of gastrointestinal drugs [26].

Nanoparticles are often used in anticancer drug delivery but the development of nanosystems for vaccine delivery is also rapidly developing. Both polymeric and liposomal particles can be used as carriers–adjuvants. Moreover, lipid-based particles were used in two vaccines against COVID—Moderna and Pfizer. Lipid-based nanoparticles provide a safe and stable structure that can be loaded with mRNA or other vaccine components. The specific structure of lipid-based particles protects their content from degradation. Adding PEG or any other coating can make the nanoparticles almost unrecognizable for immune system cells. It is also possible to add other ligands to the particle or adjust the ratio of lipid components to make it more specific for the exact tissue or organ. Overall, many authors view liposomes and other lipid-based nanoparticles as a very progressive area in vaccinology as such particles can formulate a desirable and more controlled immune response than regular vaccines [27,28,29]. When creating nanoparticles, it is necessary to factor in many aspects that affect the efficacy of the created liposomal formulation such as surface charge, size, membrane fluidity and even the way the antigen was loaded in the liposome [28]. Despite these difficulties, there are already many liposomal drugs that have been approved by the FDA, for example, Mosquirix (2015) against malaria, Marqibo (2012) against acute lymphoblastic leukemia and Onivyde (2015) against pancreatic cancer.

A 2016 study indicated that most nanoparticles that received FDA approval are either of liposomal or polymeric origin [30]. The authors point out that, due to the significant diversity of nanoparticles, in the future, trends are likely to shift towards newer types of nanoparticles [30]. A more recent 2019 review notes the emergence of a new trend in the use of nanoparticles as a way to deliver mRNAs [31]. Despite the predictions in earlier works, most of the reports since 2016 have been associated with liposomes and the field of oncology. According to other reviews, nanoparticles are mostly applied in cancer research. The authors explain the reason for this popularity with the success of FDA-approved Abraxane and Doxil, which were the majority of nanoparticles in clinical trials by the time the work was published [32]. In an update of this work in 2021 [33] authors describe more than 35 new clinical trials. They also note that 28 of these studies were focusing on lipid-based nanoparticles (25 trials out of 28 were mRNA-based vaccines) [33]. Some data indicate a further significant expansion of the spectrum of studied diseases. For instance, Crohn’s disease and multiple sclerosis might gain particular popularity as there are several drugs investigated for these diseases with some being FDA-approved, for instance, Cimzia and Copaxone [34].

Here, we provide a quantitative analysis of clinical trials focused on nanoparticles starting from 2016 to 2021. We aimed to investigate the general trends in nanoparticles research including nanoparticles to treat COVID and to compare the received information with the existing reviews. We also tried to predict most likely directions and trends in the coming years and, in connection with the current epidemiological situation, special attention was paid to the works devoted to SARS-CoV-2.

## 2. Dataset Overview

All trials included in this analysis were found on one of the largest web resources with information on various clinical trials—clinicaltrials.gov. This data source has been used for several of our previous large analysis [35,36,37]. Information about NCT number, title, status, phase, conditions, study start year and the primary completion date was collected for each analyzed clinical trial from clinicaltrials.gov. We used the term “nanoparticles” in the “Other terms” field and left the “Condition or disease” field empty. There were no criteria for inclusion/exclusion based on the gender or age of the participants or trial status. We did not search for the exact condition, except for COVID, and the trials were collected in the order in which they appeared on clinicaltrials.gov. Regarding the COVID trials, an advanced search was applied. The term “COVID-19” was used in the field “Condition or disease” and “nanoparticles”, “nanoparticle” was written in the “Other terms” field. The initial search for COVID trials identified 38 clinical trials. After manually excluding studies that were either not focusing on the treatment of COVID or did not use nanoparticles, we have included 33 clinical trials in the final list. Overall, the final dataset consisted of 486 clinical trials (including COVID trials). The studies were analyzed from the year 2002 to 2021. All the data collection and filtering were carried out manually. To check FDA approvals, we used https://www.fda.gov/drugs (accessed on 2 January 2022) or EMA (European Medicines Agency) Medicines portals. We found 13 FDA-approved drugs since 2016. We used an empirical classification of nanoparticles based on the previously published literature [17,18]. The classes included protein, polymeric, liposomal, metallic, lipid-based and other (a group with rarely mentioned nanoparticles). Among lipid-based nanoparticles, we only separated liposomal drugs due to the specificity of the dataset as most lipid-based nanoparticles were not specified. The FDA-approved drugs classification was liposomal, polymeric, inorganic, lipid-based, mucus-penetrating particles and liquid crystalline system. In this work, we reviewed only those drugs that were FDA-approved and/or had reached clinical trials.

### 2.1. Types of Nanoparticles and Drugs in Clinical Trials

Overall, we identified 486 clinical trials (including the COVID-19 trials). Out of these 486 trials, 147 were completed, 138 were recruiting, 63 active, 50 not yet recruiting, and 87 other clinical trials had unknown status (39), were terminated (27), withdrawn (18) or suspended (3). Results were available in 62 clinical trials. Out of 34 COVID-19 studies, only 1 study had published results. 

**Figure 1:**
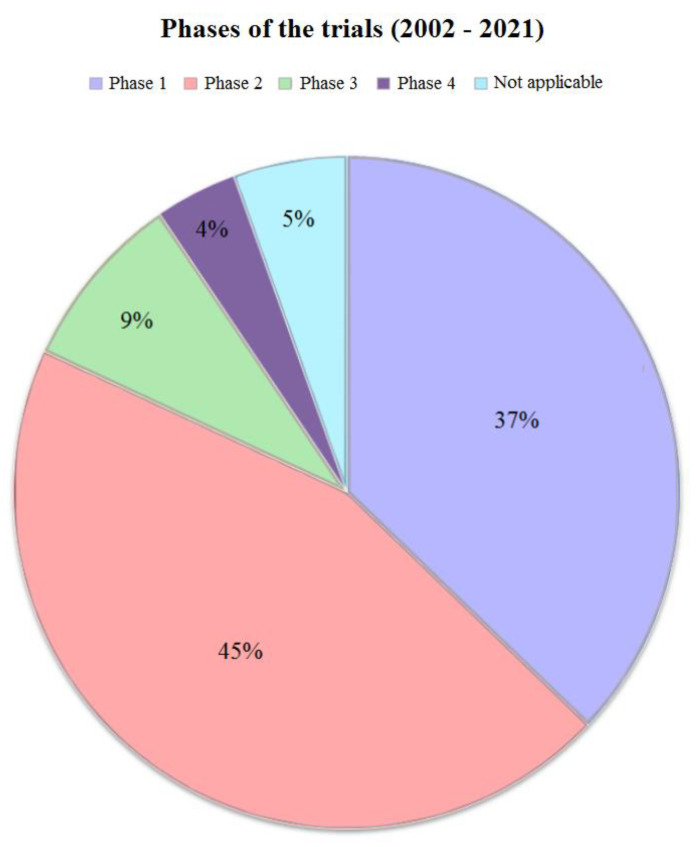
Phases of the trials. This figure provides a graphical representation of the phases of all trials (from 2002 till 2021). Most of the studies were either in first or second phase (37% and 45% respectively). Both third (9%) and fourth phases (4%) were very low among clinical trials with nanoparticles.

First, the studies were divided according to the phase (Figure 1). Most of the clinical trials were expectedly in phase 1 and phase 2. At the same time, the fourth phase was only 4% of the total sample of studies, and the number of trials in the third phase was only 8%. Upon further dividing the trials into periods from 2002 to 2016 and from 2016 to 2021, no considerable differences were found from the initial overall picture (all studies from 2002 to 2021). The year 2016 was chosen as the boundary in order to obtain the most balanced datasets for comparison.

**Figure 2:**
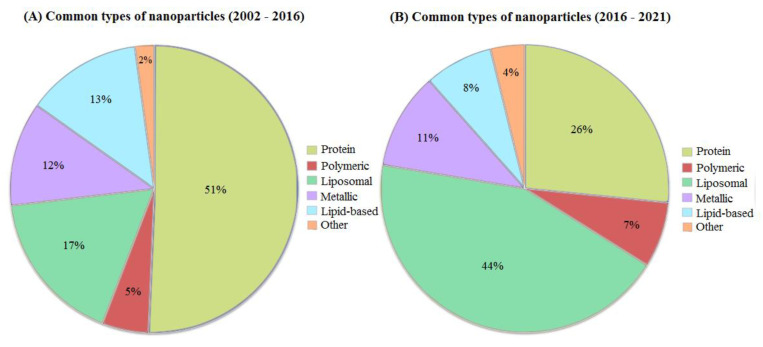
Common types of nanoparticles. This figure contains information about the types of nanoparticles used in clinical trials. (**A**) Pie chart A represents the types of nanoparticles in clinical trials from 2002 to 2016. The group other consists of carbon-based, silica-based nanoparticles and nanostructured formulations of hormones. In the 2002–2016 period, the most abundant type of nanoparticles in clinical trials was protein (51%). Liposomal formulations were the second most common but still relatively low (17%). Lipid-based nanoparticles could be encountered in 13% of all clinical trials in that period. Both metallic and polymeric formulations appeared to be scarce (12 and 5% respectively). (**B**) This part of the figure represents types of nanoparticles in trials from 2016 to 2021. The group other consists of quantum dots, micellar nanoparticles and exosomes. In comparison to 2002–2016, protein nanoparticles demonstrated a downfall (from 51% to 26%) which can be explained by an increase in liposomal drugs (from 17% to 44%). Lipid-based formulations also faced a slight decrease to 8%, while metallic and polymeric drug percentages stayed almost the same (11% and 7%, respectively).

As mentioned above, there are many different classifications of nanoparticles, depending on their type. Based on our dataset, we observed protein, polymeric, liposomal, metallic and lipid-based nanoparticles. In rare cases (no more than two studies for such types), silica-based nanoparticles, carbon-based, quantum dots and micellar nanoparticles were observed, which were combined into one group “other” (Figure 2). The most studied nanoparticles until 2016 were from group protein, trials on which occupied more than half of the total sample from 2002 to 2016 (~ 51%, ~117 clinical trials) (Figure 2A). A rather sparse group, along with other group, turned out to be polymeric nanoparticles which accounted only for 5%. Approximately the same number of studies was distributed between metallic, liposomal and lipid-based nanoparticles and did not exceed 20% of the total number of studies.

Since 2016 (Figure 2B), the situation has changed significantly. The number of trials on protein nanoparticles was equal to 26% (approximately 75 trials dedicated to protein nanoparticles) and the number of trials with liposomal drugs has become 46% out of all clinical trials since 2016 rising from 23 clinical trials in the period of 2002–2016 to 112 trials in 2016–2021. At the same time, the number of clinical trials on lipid-based nanoparticles has slightly decreased from 13% (23 trials in total) in 2002–2016 to 8% (14 trials) in 2016–2021. The percentage of clinical trials with metallic nanoparticles practically did not change in the period from 2016 to 2021, having decreased by only 1%, compared to the 2002–2016 period and being equal to 11% in 2016–2021. There is also an increase in the number of clinical trials on polymeric nanoparticles rising from 5% in 2002–2016 to 7% in 2016–2021.

**Figure 3:**
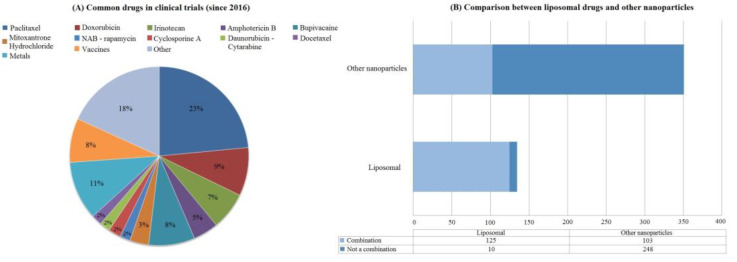
Common drugs and liposomal drugs application. The categories in (**A**) were formed based on the most common studied drugs. Mostly, each drug was mentioned individually except for the groups metals, vaccine and other. The first two groups were formed based on the heterogeneity of the drugs they comprise. The vaccine group most often consisted of mRNA vaccines, while the Metals group was dominated by various iron preparations. Others consisted of drugs that occurred no more than two times in the entire excerpt. Paclitaxel consisted of various nanoparticle types that were loaded with paclitaxel. Among them, NAB-paclitaxel was occurring the most often (57 trials out of 62 trials for paclitaxel in total). Chemotherapy drugs in general were the most common drugs in the dataset (paclitaxel—23%, doxorubicin—9%, irinotecan—7%). Different vaccines and metals were also often mentioned in clinical trials (8% and 11% respectively). Local anesthetic bupivacaine was also frequently incorporated in nanoparticle systems (8%). Obtained results could be explained by various properties that nanoparticles can have as drug carriers (for instance, site-specific drug delivery). (**B**) shows the division of clinical trials that studied liposomal drugs either individually or in combination with other regular drugs. Based on (**B**), liposomal drugs were primarily used in combination with other drugs (125 clinical trials with combinations out of a total of 135 liposomal trials). Such a phenomenon can be explained by a small amount of liposomal clinical trials in comparison with all the other types of nanoparticles. The second possible reason can be due to specific diseases (for instance, neoplasms) that require a combination of therapeutic agents and prevail in our dataset. mRNA—messenger ribonucleic acid, NAB—nanoparticle albumin-bound.

During the analysis of the collected data, it was noted that many clinical trials focused on the same drugs. We visualized drugs that are most often mentioned in clinical trials in the period 2016–2021. For simplicity, most drugs were listed separately with their names, except for the metals and vaccines groups (Figure 3A). Drugs and vaccines included in these groups were too diverse to be distinguished separately, and therefore they were combined into more general groups. The “other” group consisted of drugs that occurred no more than three times in the entire sample. There was no general trend in the “other” group, and it was fragmented. It was also concluded that it might be of great importance whether drugs in nanoparticles are used as stand-alone treatments or in combination with other non-nanoparticle drugs. It can be seen that liposomal drugs are more often combined with other drugs (Figure 3B). At the same time, other types of nanoparticles did not have such a notable difference and were used approximately equally as an independent treatment and in combination with other drugs. 

Most frequently, paclitaxel has been applied in clinical trials accounting for 23%. Specifically, it was the nanoparticle albumin-bound (NAB)—paclitaxel, which belongs to the protein nanoparticle type, that prevailed (57 trials out of 62 devoted to paclitaxel). The next most frequently mentioned group in clinical trials was the “metals” group (11%), within which various iron preparations predominated crucially. Bupivicaine, a drug used for local anesthesia, accounted for 8% of the total sample of studies. To be more specific, it was EXPAREL^®®^, a bupivacaine liposome injectable suspension. In general, almost all other drugs listed in Figure 3. Abelonged to various anticancer drugs. Vaccine preparations occupied 8% of all other drugs.

### 2.2. Different Disease Groups and Recent FDA Approvals

**Figure 4:**
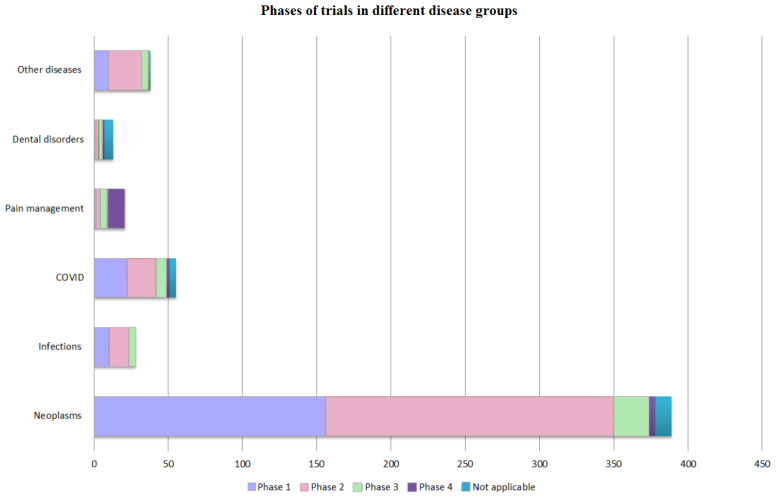
Phases of the trials in different disease groups. All studies since 2002 were categorized based on the diseases reported in the clinical trials. If several diseases were mentioned in the trial, then we divided them into separate groups of diseases. Further, each of the groups was divided based on the trials phases. The group other diseases consisted of neurological disorders, various cardiac conditions, diseases of the digestive system and rare genetic disorders. The most notable group was neoplasms which had almost 400 clinical trials. On the other hand, most of these trials were either in phase 1 or phase 2 (350 trials for both phases). The next three disease groups that also stood out were the pain management group, infections and dental disorders. In comparison to neoplasms, the number of clinical trials in these groups was very small (not more than 50 trials in each group). However, the pain management group had the biggest amount of trials that reached phase 4 (phase 4 accounted for almost half of all trials in the pain management group).

The studies were also divided according to the indications. The following groups of diseases were identified: dental disorders, neoplasms, pain management, infections, COVID and other diseases that included various neurological, cardiovascular or rare genetic disorders. Further, within each of the groups of diseases, the studies were divided according to the phase in which they were. These results are presented in Figure 4.

The prevailing part of the research was devoted to various cancer diseases. However, most of the clinical trials were either in the first or the second phase. The third and the fourth phases played a very insignificant role accounting for 24 (7.06%) and 4 (1.18%) clinical trials, respectively. The largest number of trials in phase 4 was obtained in the pain management group. This might be because most of the studies in this group were conducted using the FDA-approved EXPAREL^®®^. Phase 4 clinical trials were absent in the infections and other disease groups, which means that none of the drugs in the total sample were FDA-approved. In the COVID group, the majority of trials were in phase 1 or 2. Interestingly, the number of trials that entered the third phase of clinical trials did not differ much in all groups except for the neoplasms group.

We investigated FDA nanoparticle approvals in the same time period starting from 2016 (Table 1). Two COVID vaccines deserve special attention, as they have received a special EUA status. Under this status, the FDA could permit the use of unapproved drugs or vaccines. Some drugs have quite different FDA and EMA approval dates. For instance, Brixadi (Unite States) and Buvidal (Europe/Australia) appear to be the same drug that was approved by the FDA in 2021 and by the EMA in 2018. Arikayce, on the other hand, was approved earlier by the FDA (2018) than the EMA (2020). The same was true for Vyxeos and Shingrix (FDA—2017, EMA—2018). Some drugs mentioned in Table 1 were approved much earlier than 2016 but for different conditions. For instance, Cimzia was approved firstly in 2008 for the treatment of Crohn’s disease. We also included one of the drugs that only received a CE mark (Hensify), which means that it is yet to be evaluated for efficacy to receive FDA approval. The CE mark, however, signifies that this drug is safe for use. 

**Figure 5:**
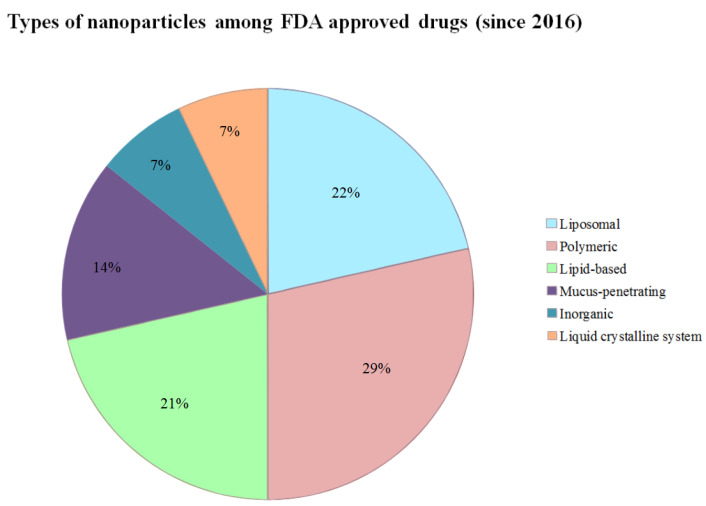
Common types of nanoparticles among FDA-approved drugs (since 2016). This figure depicts the types of nanoparticles that were used in FDA-approved medicine (since 2016). The most common were polymeric drugs (29%). The second most common were liposomal drugs (22%). However, lipid-based formulations appeared to be very close to liposomal drugs among FDA-approved medicine since 2016 (21%). Mucus-penetrating nanoparticles (14%) were applied primarily in ocular surgery to reduce post-operative inflammation. Inorganic nanoparticles and liquid crystalline system appeared only once in the table; therefore, being equal to 7% for each. This figure illustrates that polymeric, lipid-based and liposomal nanoparticles can show better results in clinical trials reaching an approval stage. On the other side, the presence of such rare and relatively new categories of nanoparticles as mucus-penetrating and liquid crystalline systems emphasizes the need for further research and development of drug-carrier systems.

All the newly approved drugs in Table 1 were divided according to their type. The following types were present: lipid-based nanoparticles, inorganic, liposomal, polymeric, mucus-penetrating nanoparticles and liquid crystalline system. The top three types were polymeric (29%), liposomal (22%) and lipid-based (21%). Mucus-penetrating nanoparticles were used in two drug preparations that mainly differed only by the concentration of the drug (Inveltys and Eysuvis, 14%) [38]. Two COVID vaccines used lipid-based nanoparticles as the carriers of immunogenic substances. These results could be seen in Figure 5.

## 3. Discussion

In this paper, we analyzed 486 clinical studies from 2002 to 2021. We see several major changes in the types of nanoparticles. A large share of the clinical trials between 2002 and 2016 were protein nanoparticles (51% out of all trials in that period) but this share has significantly declined during the 2016–2021 period (27%), while liposomal formulations (44% out of all trials) have significantly increased. However, the share of metallic nanoparticles did not change from 2002 to 2021 (from 12% in 2002–2016 to 11% in 2016–2021) (Figure 2). Clinical trials focusing on the treatment of neoplasms were increasing in numbers (340 trials) for all the years. Nanoparticles used in analgesics allow a longer duration of drug action without an increase in side effects [39]. The “Pain management” group (Figure 4) had largest number of trials in phase 4 (60%) and a notable number of trials in phase 3 (25%). Such data may indicate that the number of FDA-approved drugs in the future will increase in this particular group. Nanoparticles with paclitaxel (23% out of all trials), doxorubicin (24%) and various metals (11%) constitute the majority of all the trials (Figure 3). Vaccines and metals both were 8% out of all the clinical trials. Most of the vaccines were against COVID-19, of which the majority were lipid based. However, some other interesting examples use novel methods of nanoparticle preparation. For example, VACINA RNA MCTI CIMATEC HDT (NCT04844268) used a novel technology combining both lipid and inorganic nanoparticles (LION) [40].

The most common types of nanoparticles in the period from 2016 to 2021 were protein and liposomal nanoparticles. The result shows a significant increase in protein nanoparticles compared to the review by CL Ventola et al., in which the amount of protein nanoparticles was only 2%. At the same time, both in the 2017 analysis and in ours, liposomal particles occupy a significant part (liposomal nanoparticles occupied 30% of all investigational drugs) [41]. The other analysis from 2016 assessing the trials from 2001–2015 showed that metallic, protein, polymeric and micellar nanoparticles play an important role in clinical trials and occupy approximately the same volume. Furthermore, Figure 2 illustrates that liposomal drugs have become more common in contrast to the 2002–2016 (from 17% to 44%) and 2001–2015 periods [40]. 

The reason why protein and liposomal nanoparticles gained such a large percentage in our results (26% and 44%) may be related to the specific diseases for which such drugs are used. Protein nanoparticles are most often used for the preparation of anticancer drugs (paclitaxel, doxorubicin, etc.). Protein nanoparticles also offer an opportunity for the oral administration of peptides loaded in nanoparticles, for example, NPs loaded with insulin [42]. The reason for the growth of liposomal drugs is more complex. One of the possible directions is the use of liposomal formulations as a replacement for existing drugs to reduce toxic effects (for example, Doxil significantly reduces cardiotoxicity compared to conventional doxorubicin) [43]. Some authors state that liposomal medicines may be a promising choice in the treatment of neoplasms. Although there are several serious problems, such as reproducibility, the authors indicate that overcoming these issues is certainly possible [15]. The recently approved Onivyde, which can be applied for the treatment of pancreatic cancer, proves that the existing issues with liposomal drugs could be successfully addressed. Another reason for such a large number of clinical trials on liposomal drugs is their use for vaccine delivery. Serving either as vaccine carriers or adjuvant platforms, they could potentiate the immune response. Moreover, they could be modified in a way that they become specific to only certain types of cells, for example, dendritic cells [44]. Liposomes also tend to cause fewer toxic effects than regular drugs, which could be perceived as a propitious area in vaccinology, especially during the COVID-19 period [45]. Overall, research shows that liposomes gained several merits besides the low risk of developing toxic effects, relating them to often being biologically inert, being biodegradable and having the ability to reduce the frequency of drug intake as well as the actual volume of active substances [46].

The high number of trials with bupivacaine may appear as an intriguing result. There is a growing demand for prolonged anesthetics, especially for local treatment. This relates to the fact that bupivacaine has low molecular weight and fast absorption that leads to a short duration of their effects. One of the advantages of using nanoparticles as a drug delivery tool is the lengthening of the drug release time which, alongside the reduction of systemic toxicity, was implemented for bupivacaine [39,47]. The 340 clinical trials focused on the treatment of various neoplasms may be the explanation for such a high percentage of paclitaxel and doxorubicin, both of which are anticancer drugs. The notable interest in anticancer drugs has led to the fact that the number of clinical trials in other areas of medicine was scarce in comparison. For instance, there were only seven clinical trials on cardiovascular disorders, four on neurological and twelve on dental indications Recent works indicate that nanoparticles may appear as a solution to many cardiovascular disorders due to the development of various administration approaches. For example, loading nanoparticles in immune cells as Trojan horses showed better results than regular intravenous administration [48,49]. Despite the growing number of reviews and experimental studies on the use of nanoparticles both for the treatment of heart diseases and in other areas, they all agree that more clinical trials are required to reach a more convincing conclusion [48,49,50,51]. Taking into account the current epidemiological situation, there is strong interest in developing nanoparticles for the treatment of different infections, such as for COVID-19. 

The most prevalent categories of FDA-approved drugs since 2016 were polymeric (29%), liposomal (22%) and lipid-based (21%). Liposomal drugs were also occupying 44% of all drug types in investigational drugs in the period from 2016 to 2021. However, polymeric nanoparticles only stand for 7% of investigational drugs (Figure 2). Polymeric nanoparticles are a promising approach in vaccine and antibiotics delivery as well as cancer therapy due to their favorable characteristics that comprise wide structure variety and also comparatively uncomplicated elaboration and design. Several polymeric nanoparticles tend to show better results when compared with regular administration [23]. Considering that liposomal nanoparticles are sometimes grouped with all other lipid-based formulations, the overall percentage among FDA-approved nanoparticle drugs would be 43% (Figure 5). Therefore, lipid-based nanoparticles (especially, liposomal) appeared to be the most common applied drugs in both clinical trials and FDA-approved drugs since 2016 according to our data (52% of lipid-based substances in clinical trials and 43% among FDA-approved drugs). The predominance of liposomes among lipid nanoparticles is also indicated in some of the recent reviews [52,53,54]. Liposomes have shown remarkable results due to their high bioavailability and relatively low immunogenicity [55]. However, some authors also note the existing gap between the number of clinical lipid formulations and those that have reached the market, which might explain a small amount of FDA-approved lipid formulations (six drugs, Table 1) [28]. 

We expect a further increase in the number of trials for polymeric and lipid-based nanoparticles. Both ongoing clinical trials and FDA-approved drugs suggest the emergence of new types of nanoparticles that need to be further investigated. Micellar, carbon-based and quantum dot nanoparticles (only 3% of them all in Figure 2) are types that may see an increased number of trials. This might be due to some of them being new interventions, for instance, carbon dots were discovered only in 2004. Nevertheless, recent experimental studies showed that these nanoparticles could perform good results in terms of cancer targeting [56,57,58]. It has been pointed out that micellar nanoparticles may contribute to overcome drug resistance in cancer patients [59,60,61,62].

## 4. Materials and Methods

Clinical trials on nanoparticles are very diversified, being applied in many medical fields using different vocabulary. It is therefore difficult to choose the most appropriate search terms to avoid being biased towards a specific medical area. Therefore, only the words “nanoparticle” and “nanoparticles” were used as general search terms beyond COVID-19. We did not search separately for different types of nanoparticles, such as liposomes or protein-based constructs. The search was performed only using the clinicaltrials.gov database as this database is known to be well structured and is one of the largest registries of clinical trials (the largest in the United States). We thus acknowledge that our results may not reflect all different countries and regions. The strength of this work, however, is that every clinical trial analyzed was checked manually, minimalizing the risk of inappropriate study inclusion. Special attention and advanced searches were applied towards COVID-19 trials in order to achieve the most precise results. We did not focus on very specific areas of medicine with the intention to illustrate general trends in nanoparticles to make this analysis appeal to a wider variety of specialists. However, the raw data are available for further analysis upon request to the authors.

## 5. Conclusions

There are approximately 64 nanoparticles that have already been FDA approved. We see that previously known drugs are receiving approvals for new indication beyond the developing new unique interventions. Such characteristics as biodegradability, different coating and antigens that can be bound to the nanoparticle and low toxic effects are to a certain degree common for all types of nanosystems. These traits make nanointerventions more desirable compared to regular administration. Although nanoparticles are mostly applied in the field of oncology, growth trends are notable in other areas of medicine. Dental disorders, pain management and infections were the three groups with a small but rapidly growing number of trials. Nanoparticles have become more actively viewed as delivery systems or adjuvants for vaccines being then used in Moderna and Pfizer vaccines. The outlook for polymeric, liposomal and lipid-based nanoparticles seems to be the most promising as these were the top categories among newly FDA-approved drugs. We suggest that, due to the rapid development of technology and medicine, there could be a future increase in studies on rare nanoparticle types such as micellar, exosomes and quantum dots.

## Figures and Tables

**Table 1:** FDA-approved drugs since 2016. PLGA—poly-(d,l-lactic acid-co-glycolic acid), OUD—opioid use disorder, TTR—transthyretin, MAC—Mycobacterium Avium Complex, siRNA—small interfering RNA, ESA—erythropoiesis-stimulating agent, mRNA—messenger ribonucleic acid.

Name	Particle Type/Drug	Approved Application/ Indication	Approval (Year)	Investigated Application/ Indication	Drug Target
CPX-351/Vyxeos	Liposomal formulation of cytarabine and daunorubicin	Acute myeloid leukemia	FDA (2017) EMA (2018)	Various leukemias	Anthracycline topoisomerase inhibitor (daunorubicin) Nucleoside metabolic inhibitor (cytarabine)
FX006/Zilretta	PLGA hydrogel/Triamcinolone acetonide extended-release injectable suspension	Osteoarthritis pain syndrome	FDA (2017)	Adhesive Capsulitis Frozen Shoulder	Glucocorticoid responsive elements Phospholipase A2 inhibition Inhibition of NF-kB
Recombinant zoster vaccine (RZV, Shingrix)	Liposomal nanoparticles/Recombinant glycoprotein E with an adjuvant system	Shingles	FDA (2017) 2021—for immunocompromised adults EMA (2018)	-	CD4^+^ response (primarily) Anti-glycoprotein E antibodies
Rebinyn/Refixia	Polymeric nanoparticles/Coagulation Factor IX (Recombinant), GlycoPEGylated	Hemophilia B	FDA (2017) EMA (2017)	-	Replacement of Coagulation Factor IX
Sublocade (buprenorphine)	PLGA nanoparticles/Once-monthly injectable formulation	Opioid use disorder (OUD)	FDA (2017)	Evaluating safety in patients with Sickle Cell Disease	Partial mu-agonist, kappa antagonist
Brixadi/Buvidal (buprenorphine)	Liquid crystalline system	Opioid use disorder (OUD)	FDA (2021) EMA (2018)	Evaluating safety in patients with Sickle Cell Disease	Partial mu-agonist, kappa antagonist
ALN-TTR02/Patisiran/Onpattro	Lipid nanoparticles with siRNA	Transthyretin (TTR)-mediated amyloidosis	FDA (2018) EMA (2018)	Transthyretin (TTR)-mediated amyloidosis with cardiomyopathy/polyneuropathies	Transthyretin (TTR) messenger RNA
ALIS/Arikayce	Liposomal nanoparticles/Amikacin sulfate	Mycobacterium Avium Complex (MAC) Lung Disease	FDA (2018) EMA (2020)	Cystic Fibrosis	Bacterial 30S ribosomal subunits
CDP870/Cimzia	Polymeric nanoparticles/Certolizumab pegol	Moderate-to-severe plaque psoriasis/Non-radiographic axial spondyloarthritis	FDA (2018)/FDA (2019)	Evaluating safety and efficacy in pregnancy Lung cancer (stages II–IV)	TNF-α
B03XA03/Mircera	Polymeric nanoparticles/Erythropoiesis-stimulating agent (ESA)	Anemia associated with chronic kidney disease in patients from 5 to 17 years old on hemodialysis	FDA (2018)	-	Erythropoietin receptor
KPI-121 1%/Inveltys	Mucus-penetrating particles/Loteprednol etabonate ophthalmic nanosuspension	Post-operative inflammation and pain following ocular surgery	FDA (2018)	Vernal Keratoconjunctivitis Keratoconus Blepharitis	Glucocorticoid responsive elements Phospholipase A2 inhibition Inhibition of NF-kB
KPI-121 0.25%/Eysuvis	Mucus-penetrating particles/Loteprednol etabonate ophthalmic nanosuspension	Short-term treatment of dry eye disease	FDA (2020)	Prevention of cornea transplant rejection	Glucocorticoid responsive elements Phospholipase A2 inhibition Inhibition of NF-kB
NBTXR3/Hensify	Inorganic/Hafnium oxide nanoparticles	Locally advanced squamous cell carcinoma	CE Mark (2019)	Locally advanced soft tissue sarcoma	Cancer cells
mRNA-1273/Moderna/Spikevax	Lipid nanoparticles/mRNA-based vaccine	COVID-19 Immunisation	FDA(2020)—EUA EMA (2022)—Standard marketing authorisation	Evaluating immunogenicity in various groups	CD4^+^, CD8^+^ response Neutralizing antibodies production
Pfizer-BioNTech/Comirnaty	Lipid nanoparticles/mRNA vaccine	COVID-19 Immunisation	FDA (2020)—EUA EMA (2022)—Standard marketing authorisation	Evaluating immunogenicity in various groups	CD4^+^, CD8^+^ response Neutralizing antibodies production

## Data Availability

Publicly available datasets were analyzed in this study. This data can be found here: https://www.fda.gov/drugs.

## List of Abbreviations

AIDS	acquired immunodeficiency syndrome
CE Mark	European Conformity Mark
EMA	European Medicines Agency
ESA	erythropoiesis-stimulating agent
EUA	emergency use authorization
FDA	food and drug administration
LION	lipid and inorganic nanoparticles
MAC	Mycobacterium Avium Complex
mRNA	messenger ribonucleic acid
NAB	nanoparticle albumin-bound
NP	nanoparticle
OUD	opioid use disorder
PEG	polyethylene glycol
PLGA	poly-(d,l-lactic acid-co-glycolic acid)
siRNA	small interfering RNA
TTR	transthyretin

## References

[1] Bayda S., Adeel M., Tuccinardi T., Cordani M., Rizzolio F. (2020). The History of Nanoscience and Nanotechnology: From Chemical–Physical Applications to Nanomedicine. Molecules.

[2] Yamada Y., Sato Y., Nakamura T., Harashima H. (2022). Innovative cancer nanomedicine based on immunology, gene editing, intracellular trafficking control. J. Control. Release.

[3] De Lázaro I., Mooney D.J. (2021). Obstacles and opportunities in a forward vision for cancer nanomedicine. Nat. Mater..

[4] Bhatia S.N., Chen X., Dobrovolskaia M.A., Lammers T. (2022). Cancer nanomedicine. Nat. Rev. Cancer.

[5] Van der Meel R., Sulheim E., Shi Y., Kiessling F., Mulder W.J., Lammers T. (2019). Smart cancer nanomedicine. Nat. Nanotechnol..

[6] Reimbursement Team (2021). Daunorubicin and Cytarabine Liposome (Vyxeos). Can. J. Health Technol..

[7] Germain M., Caputo F., Metcalfe S., Tosi G., Spring K., Åslund A.K., Pottier A., Schiffelers R., Ceccaldi A., Schmid R. (2020). Delivering the power of nanomedicine to patients today. J. Control. Release.

[8] Jacoby M.A., Sallman D.A., Scott B.L., Haney M., Wan F., DiPersio J.F., Abboud R., Stockerl-Goldstein K.E., Komrokji R.S., Schroeder M.A. (2021). A Pilot Study of CPX-351 (Vyxeos©) for Transplant Eligible, Higher Risk Patients with Myelodys-plastic Syndrome. Blood.

[9] Hou T., Sana S.S., Li H., Wang X., Wang Q., Boya V.K.N., Vadde R., Kumar R., Kumbhakar D.V., Zhang Z. (2022). Development of Plant Protein Derived Tri Angular Shaped Nano Zinc Oxide Particles with Inherent Antibacterial and Neurotoxicity Properties. Pharmaceutics.

[10] Liu W., Chen B., Zheng H., Xing Y., Chen G., Zhou P., Qian L., Min Y. (2021). Advances of Nanomedicine in Radiotherapy. Pharmaceutics.

[11] Irvine D.J., Dane E.L. (2020). Enhancing cancer immunotherapy with nanomedicine. Nat. Rev. Immunol..

[12] Van Leent M.M., Priem B., Schrijver D.P., de Dreu A., Hofstraat S.R., Zwolsman R., Beldman T.J., Netea M.G., Mulder W.J. (2022). Regulating trained immunity with nanomedicine. Nat. Rev. Mater..

[13] Douek M., Klaase J., Monypenny I., Kothari A., Zechmeister K., Brown D., Wyld L., Drew P., Garmo H., On behalf of the SentiMAG Trialists Group (2013). Sentinel Node Biopsy Using a Magnetic Tracer Versus Standard Technique: The SentiMAG Multicentre Trial. Ann. Surg. Oncol..

[14] Sharifianjazi F., Irani M., Esmaeilkhanian A., Bazli L., Asl M.S., Jang H.W., Kim S.Y., Ramakrishna S., Shokouhimehr M., Varma R.S. (2021). Polymer incorporated magnetic nanoparticles: Applications for magnetoresponsive targeted drug delivery. Mater. Sci. Eng. B.

[15] Tran S., DeGiovanni P.-J., Piel B., Rai P. (2017). Cancer nanomedicine: A review of recent success in drug delivery. Clin. Transl. Med..

[16] Mirza A.Z., Siddiqui F.A. (2014). Nanomedicine and drug delivery: A mini review. Int. Nano Lett..

[17] Ijaz I., Gilani E., Nazir A., Bukhari A. (2020). Detail review on chemical, physical and green synthesis, classification, characterizations and applications of nanoparticles. Green Chem. Lett. Rev..

[18] Ealia S.A.M., Saravanakumar M.P. (2017). A review on the classification, characterisation, synthesis of nanoparticles and their application. IOP Conf. Ser. Mater. Sci. Eng..

[19] Zahin N., Anwar R., Tewari D., Kabir M.T., Sajid A., Mathew B., Uddin M.S., Aleya L., Abdel-Daim M.M. (2019). Nanoparticles and its biomedical applications in health and diseases: Special focus on drug delivery. Environ. Sci. Pollut. Res..

[20] Nene A., Galluzzi M., Hongrong L., Somani P., Ramakrishna S., Yu X.-F. (2021). Synthetic preparations and atomic scale engineering of silver nanoparticles for biomedical applications. Nanoscale.

[21] Ajinkya N., Yu X., Kaithal P., Luo H., Somani P., Ramakrishna S. (2020). Magnetic Iron Oxide Nanoparticle (IONP) Synthesis to Applications: Present and Future. Materials.

[22] Wu K., Su D., Liu J., Saha R., Wang J.-P. (2019). Magnetic nanoparticles in nanomedicine: A review of recent advances. Nanotechnology.

[23] Khalid M., El-Sawy H.S. (2017). Polymeric nanoparticles: Promising platform for drug delivery. Int. J. Pharm..

[24] Large D.E., Abdelmessih R.G., Fink E.A., Auguste D.T. (2021). Liposome composition in drug delivery design, synthesis, characterization, and clinical application. Adv. Drug Deliv. Rev..

[25] Beltrán-Gracia E., López-Camacho A., Higuera-Ciapara I., Velázquez-Fernández J.B., Vallejo-Cardona A.A. (2019). Nanomedicine review: Clinical developments in liposomal applications. Cancer Nanotechnol..

[26] Mamidi N., Delgadillo R.M.V. (2021). Design, fabrication and drug release potential of dual stimu-li-responsive composite hydrogel nanoparticle interfaces. Colloids Surf. B Biointerfaces.

[27] Kheirollahpour M., Mehrabi M., Dounighi N.M., Mohammadi M., Masoudi A. (2020). Nanoparticles and Vaccine Development. Pharm. Nanotechnol..

[28] Thi T., Suys E., Lee J., Nguyen D., Park K., Truong N. (2021). Lipid-Based Nanoparticles in the Clinic and Clinical Trials: From Cancer Nanomedicine to COVID-19 Vaccines. Vaccines.

[29] Chatzikleanthous D., O’Hagan D.T., Adamo R. (2021). Lipid-Based Nanoparticles for Delivery of Vaccine Adjuvants and Antigens: Toward Multicomponent Vaccines. Mol. Pharm..

[30] Bobo D., Robinson K.J., Islam J., Thurecht K.J., Corrie S.R. (2016). Nanoparticle-Based Medicines: A Review of FDA-Approved Materials and Clinical Trials to Date. Pharm. Res..

[31] Anselmo A.C., Mitragotri S. (2019). Nanoparticles in the clinic: An update. Bioeng. Transl. Med..

[32] Anselmo A.C., Mitragotri S. (2021). Nanoparticles in the clinic: An update post COVID-19 vaccines. Bioeng. Transl. Med..

[33] Anselmo A.C., Mitragotri S. (2016). Nanoparticles in the clinic. Bioeng. Transl. Med..

[34] Kumar B., Jalodia K., Kumar P., Gautam H.K. (2017). Recent advances in nanoparticle-mediated drug delivery. J. Drug Deliv. Sci. Technol..

[35] Attwood M.M., Fabbro D., Sokolov A.V., Knapp S., Schiöth H.B. (2021). Trends in kinase drug discovery: Targets, indications and inhibitor design. Nat. Rev. Drug Discov..

[36] Attwood M.M., Jonsson J., Rask-Andersen M., Schiöth H.B. (2020). Soluble ligands as drug targets. Nat. Rev. Drug Discov..

[37] Dambrova M., Makrecka-Kuka M., Kuka J., Vilskersts R., Nordberg D., Attwood M.M., Smesny S., Sen Z.D., Guo A.C., Oler E. (2022). Acylcarnitines: Nomenclature, Biomarkers, Therapeutic Potential, Drug Targets, and Clinical Trials. Pharmacol. Rev..

[38] Dang D.H., Riaz K.M., Karamichos D. (2022). Treatment of Non-Infectious Corneal Injury: Review of Di-agnostic Agents, Therapeutic Medications, and Future Targets. Drugs.

[39] Ma P., Li T., Xing H., Wang S., Sun Y., Sheng X., Wang K. (2017). Local anesthetic effects of bupivacaine loaded lipid-polymer hybrid nanoparticles: In vitro and in vivo evaluation. Biomed. Pharmacother..

[40] Hawman D.W., Meade-White K., Archer J., Leventhal S.S., Wilson D., Shaia C., Randall S., Khandhar A.P., Krieger K., Hsiang T.Y. (2022). SARS-CoV2 variant-specific replicating RNA vaccines protect from disease following challenge with heterologous variants of concern. Elife.

[41] Ventola C.L. (2017). Progress in nanomedicine: Approved and investigational nanodrugs. Pharm. Ther..

[42] Verma D., Gulati N., Kaul S., Mukherjee S., Nagaich U. (2018). Protein Based Nanostructures for Drug Delivery. J. Pharm..

[43] Safra T., Muggia F., Jeffers S., Tsao-Wei D.D., Groshen S., Lyass O., Henderson R., Berry G., Gabizon A. (2000). Pegylated liposomal doxorubicin (doxil): Reduced clinical cardiotoxicity in patients reaching or exceeding cu-mulative doses of 500 mg/m^2^. Ann. Oncol..

[44] Unger W.W., van Beelen A.J., Bruijns S.C., Joshi M., Fehres C.M., van Bloois L., Verstege M.I., Ambrosini M., Kalay H., Nazmi K. (2012). Glycan-modified liposomes boost CD4+ and CD8+ T-cell responses by targeting DC-SIGN on dendritic cells. J. Control. Release.

[45] Yu R., Mai Y., Zhao Y., Hou Y., Liu Y., Yang J. (2018). Targeting strategies of liposomal subunit vaccine delivery systems to improve vaccine efficacy. J. Drug Target..

[46] Miere F., Fritea L., Cavalu S., Vicas S.I. (2020). Formulation, characterisation and advantages of using liposomes in multiple therapies. Pharmacophore.

[47] De Paula E., Cereda C.M., Fraceto L.F., de Araujo D.R., Franz-Montan M., Tofoli G.R., Ranali J., Volpato M.C., Groppo F.C. (2012). Micro and nanosystems for delivering local anesthetics. Expert Opin. Drug Deliv..

[48] Smith B.R. (2021). Nanotherapeutics for cardiovascular disease. Nat. Rev. Cardiol..

[49] Cheraghi M., Negahdari B., Daraee H., Eatemadi A. (2017). Heart targeted nanoliposomal/nanoparticles drug delivery: An updated review. Biomed. Pharmacother..

[50] Zhu C., Ma J., Ji Z., Shen J., Wang Q. (2021). Recent Advances of Cell Membrane Coated Nanoparticles in Treating Cardiovascular Disorders. Molecules.

[51] Chandarana M., Curtis A., Hoskins C. (2018). The use of nanotechnology in cardiovascular disease. Appl. Nanosci..

[52] García-Pinel B., Porras-Alcalá C., Ortega-Rodríguez A., Sarabia F., Prados J., Melguizo C., López-Romero J.M. (2019). Lipid-Based Nanoparticles: Application and Recent Advances in Cancer Treatment. Nanomaterials.

[53] Samimi S., Maghsoudnia N., Eftekhari R.B., Dorkoosh F. (2019). Lipid-based nanoparticles for drug delivery systems. Charact. Biol. Nanomater. Drug Deliv..

[54] Ben Shimon M., Shapira S., Seni J., Arber N. (2022). The Big Potential of Small Particles: Lipid-Based Nanoparticles and Exosomes in Vaccination. Vaccines.

[55] Liang W., Dong Y., Shao R., Zhang S., Wu X., Huang X. (2022). Application of nanoparticles in drug delivery for the treatment of osteosarcoma: Focussing on the lipo-somes. J. Drug Target..

[56] Jana P., Dev A. (2022). Carbon quantum dots: A promising nanocarrier for bioimaging and drug delivery in cancer. Mater. Today Commun..

[57] Abdelhamid H.N. (2022). Quantum dots hybrid systems for drug delivery. Hybrid Nanomaterials for Drug Delivery.

[58] Kotta S., Aldawsari H.M., Badr-Eldin S.M., Nair A.B., Yt K. (2022). Progress in Polymeric Micelles for Drug Delivery Applications. Pharmaceutics.

[59] Ruzycka-Ayoush M., Kowalik P., Kowalczyk A., Bujak P., Nowicka A.M., Wojewodzka M., Kruszewski M., Grudzinski I.P. (2021). Quantum dots as targeted doxorubicin drug delivery nanosystems in human lung cancer cells. Cancer Nanotechnol..

[60] Pardo J., Peng Z., Leblanc R.M. (2018). Cancer Targeting and Drug Delivery Using Carbon-Based Quantum Dots and Nanotubes. Molecules.

[61] Qu Y., Chu B., Shi K., Peng J., Qian Z. (2017). Recent Progress in Functional Micellar Carriers with Intrinsic Therapeutic Activities for Anticancer Drug Delivery. J. Biomed. Nanotechnol..

[62] Atanase L.I. (2021). Micellar Drug Delivery Systems Based on Natural Biopolymers. Polymers.

